# Reinvigorating postpartum intrauterine contraceptive device use in Pakistan: an observational assessment of competency-based training of health providers using low-cost simulation models

**DOI:** 10.1186/s12909-019-1683-y

**Published:** 2019-07-15

**Authors:** Zonobia Zafar, Hammad Habib, Adrienne Kols, Fauzia Assad, Enriquito R. Lu, Anne Schuster

**Affiliations:** 1 Jhpiego Pakistan, 1st Floor, 85 – East Kamran Center Jinnah Avenue, Blue Area, Islamabad, Pakistan; 2 Common Unit for Managing the Global Fund, Ministry of National Health Services Regulations and Coordination, NIH, 1st Floor, Chak Shahzad, Islamabad, Pakistan; 3 Jhpiego, 1615 Thames Street, Baltimore, MD 21231 USA

**Keywords:** Postpartum family planning, Intrauterine contraceptive device, Anatomic models, Task shifting, Pakistan

## Abstract

**Background:**

Improved training approaches have the potential to overcome barriers to the use of postpartum intrauterine devices (PPIUDs) in Pakistan, including a shortage of female providers who are able to insert the device. This study assessed the effectiveness and acceptability of a competency-based onsite training approach that employed a newly developed anatomic model (the Mama-U) to train doctors and midwives on postpartum family planning (PPFP) and the insertion of PPIUDs.

**Methods:**

An observational, mixed methods study conducted training evaluations and knowledge and skills assessments with 11 trainers and 88 doctors and midwives who participated in eight PPIUD training sessions. Two months later, follow-up interviews and clinical assessments were conducted with 20 providers, and interviews and a focus group discussion were conducted with 85 married women who received a PPIUD from a trained provider.

**Results:**

The training significantly improved provider knowledge (*p* < 0.001), and follow-up assessments showed that clinical skills were retained for at least two months post-training. After training, 81.8% of providers were confident in their ability to provide PPIUD services, and midwives and doctors had similar PPIUD insertion skills. However, midwives were more likely than doctors to meet all 10 key requirements during PPIUD counseling sessions (63.9% versus 13.3%, *p* = 0.004). Providers found the Mama-U model to be a useful tool for client counseling as well as training and skills practice, and clients agreed. Trainers identified the low cost, light weight, and portability of the Mama-U model as advantages over the conventional training model and noted that its abstract shape reduced embarrassment among trainers, providers, and clients.

**Conclusions:**

Competency-based training with the Mama-U model can improve the quality of PPIUD counseling and PPIUD insertion services and has the potential to extend PPFP/PPIUD service delivery to midwives working in rural Pakistan. The portable, low-cost Mama-U permits onsite, on-the-job PPIUD insertion training that is tailored to the local setting; it is also well suited for the continuing practice that providers need to maintain their skills. Further research is needed to confirm the usefulness and cost-effectiveness of the Mama-U at scale and in other settings.

**Electronic supplementary material:**

The online version of this article (10.1186/s12909-019-1683-y) contains supplementary material, which is available to authorized users.

## Background

The World Health Organization (WHO) recommends a minimum interpregnancy interval of 24 months following a live birth. However, the results of the Pakistan Demographic and Health Surveys indicate that many women become pregnant before this. From 2006-07 to 2012–13, the percentage of births occurring within 24 months of a live birth in Pakistan increased from 34 to 37% [1, 2]. This trend may be related to Pakistan’s high unmet need for family planning, 64%, during the first year postpartum [3]. Postpartum family planning (PPFP) uptake appears especially low for long-acting methods, such as postpartum intrauterine devices (PPIUDs) [3, 4].

Strengthening PPFP services – especially for PPIUDs – has the potential to reduce unmet need and improve maternal and newborn health outcomes in Pakistan. PPIUDs have many advantages: they offer safe, effective, long-acting contraception, can be inserted during the 48 h following delivery, are easily incorporated into post-delivery care, do not affect breastfeeding, are safe for HIV-positive women, and are immediately reversible if a couple desires a return to fertility [5]. However, only 2.3% of married women of reproductive age in Pakistan currently use an intrauterine device (IUD) for contraception [2]. Although IUDs have been available in Pakistan for decades, recent surveys of married women in Punjab found that only around one-third had heard of IUDs [6, 7].

Low PPIUD initiation rates may be due to many factors, including a lack of provider confidence and client mistrust of the method, as well as systemic challenges like contraceptive supply-chain management [8]. In Punjab, only 17% of Basic Health Units were fully functional for providing preventive maternal, newborn, and child health services in 2011 [9]. Access to high quality PPFP services, especially those requiring strong clinical knowledge and skills, is also complicated by cultural norms surrounding women’s health services in Pakistan. In many settings in that country, women can only receive medical treatment from other women, and many women will not consider accepting a contraceptive method if their husband does not support family planning [10].

Although female doctors are best equipped to provide PPFP services in Pakistan, they are scarce. Typically, the first and, in rural areas, often the only source of health care for many women are midwives who, for the purposes of this paper, are defined as including Community Midwives (CMWs), facility-based nurses, Family Welfare Workers (FWWs), and Lady Health Visitors (LHVs); in addition, Lady Health Workers (LHWs) provide non-clinical family planning and other health counseling services at the community level. However, Pakistan is currently experiencing difficulty training and retaining highly skilled female health workers [11]. Nationwide, there are only 12 doctors, nurses, and midwives per 10,000 people, well below the WHO-recognized threshold of 23 [12], and most doctors work at tertiary care hospitals and in urban areas. Given the shortage of health workers, it is essential that all health care providers who are permitted to offer clinical care to women – especially female providers – be trained to offer PPIUD services. However, this is not currently standard practice. Female patients in Pakistan often prefer to see Woman Medical Officers (WMOs), who are female doctors eligible to provide clinical care to women according to national guidelines. However, as of 2010–11, only 83 WMOs had been trained in family planning counselling and 23 in clinical family planning delivery nationwide; they represented only 8 and 2%, respectively, of all WMOs who received any training through the national Maternal, Newborn and Child Health Programme [9].

Additional challenges complicate PPIUD training in Pakistan. National clinical guidelines for PPIUD insertion were only published in 2011, and competency-based training for PPIUD insertion is lacking. Pakistan’s midwifery curriculum includes 12 h for theory and 54 h for clinical practice in family planning, which includes IUD counseling and insertion. In medical schools, students learn clinical and counseling skills related to all family planning methods, including IUDs, during two-month postings in obstetrics and gynecology units. All IUD training opportunities rely on anatomic models that are expensive, difficult to transport, and not durable enough to withstand the wear and tear of repeated use. As a result, training on PPIUD insertion is often conducted in large groups at centrally located training facilities, which require health workers to travel long distances and may leave peripheral facilities serving rural populations without providers for long periods. Furthermore, the cost, size, and bulkiness of current anatomic models precludes newly trained providers from carrying them back to service delivery sites for self-paced, on-the-job practice, which has proven to be an effective tool for expanding PPIUD insertion responsibilities to midwives [13]. As a result, fewer providers are trained on PPIUD insertion, and those who are trained are unable to maintain their skills [14].

To expand access to PPFP services, Jhpiego, along with Laerdal Medical, developed a new strategy for training doctors, nurses, and community health workers on PPFP service delivery, including PPIUD insertion. Jhpiego is an international, non-profit health organization affiliated with Johns Hopkins University that has supported programs in more than 155 countries, including Pakistan, for over four decades. The strategy introduced through Jhpiego’s collaboration with Laerdal Medical relies on a proven competency-based, blended learning approach and a portable, cost-effective training model called the Mama-U. The implementation research described in this paper assessed the effectiveness and acceptability of this strategy in Pakistan in collaboration with the Saving Lives at Birth Partnership. The assessment focused on three primary research questions:

1. Did competency-based training using the Mama-U model give doctors and midwives the essential knowledge and skills necessary to provide good quality PPIUD services?
2. Did providers maintain their skills for two months after training?
3. Was the Mama-U model acceptable to providers and practical for training all cadres in a resource-poor setting?

## Methods

### Study design and sample

An observational, mixed methods study was conducted in two phases between June 2012 and September 2013: first during PPFP/PPIUD clinical trainings with the Mama-U model at tertiary and secondary public and private health facilities and then approximately two months afterwards during post-training follow-up visits to these same facilities. During the first phase, data were collected from the 11 trainers who led the PPIUD training sessions and the 88 doctors and midwives who attended them.

**Fig. 1:**
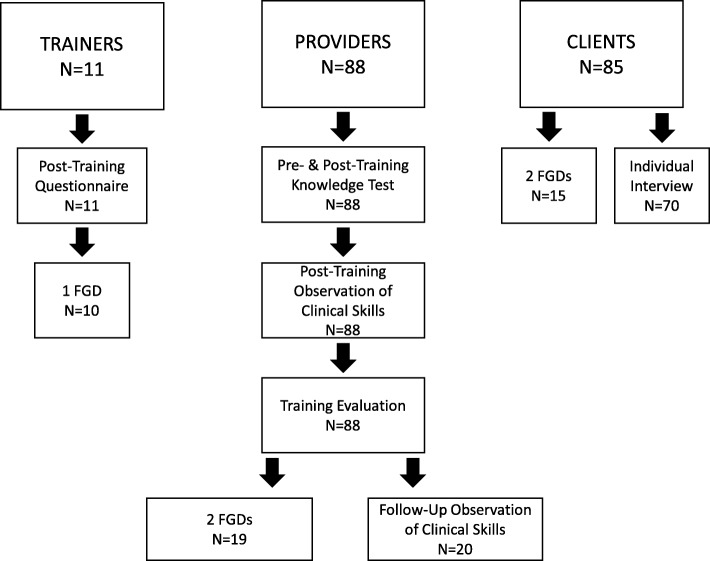
Study Participants and Data Sources

During the second phase, further data were collected from 20 of the providers who had consented to follow-up assessments and were present at study facilities on the day of follow-up data collection. Data were also collected from a convenience sample of 85 married women aged 18–49 who opted for and received a PPIUD from a provider trained by the study and were present at a study facility during data collection. Seventy clients were interviewed, and 15 participated in two focus group discussions (FGDs). No more than five providers per facility were included in the follow-up and no more than 20 clients per facility were interviewed in order to assure good representation from all sites in the sample. The sampling and data collection approach is outlined in Fig. 1.

Verbal consent was obtained from all participants before enrollment in the study and documented in study records. Copies of recruitment and consent materials were provided to each participant. All data collectors were trained in obtaining informed consent during a one-week workshop. Consent discussions were held in both English and Urdu, depending on the participant’s preference. These consent procedures were approved by the Johns Hopkins Bloomberg School of Public Health Institutional Review Board (IRB 00004782) and by the National Bioethics Committee of Pakistan (ref: 4–87/13/NBC-119/RDC/4915).

### Description of the intervention

The intervention sought to improve access to PPIUDs by using a proven competency-based training approach together with the newly developed anatomic model to train midwives, including those working predominantly in the community, and doctors/WMOs on the method. This training expanded the roles of community-based midwives and nurses, who normally refer clients to doctors for clinically administered methods of family planning. This type of task shifting has been proven to improve the utilization of a variety of health care services in India, Nepal, and Nigeria, among others [15].

Jhpiego collaborated with Laerdal to develop a portable, low-cost training simulation model for PPIUD insertion based on Laerdal’s effective Mama Natalie platform [16]. The new Mama-U model simulates a postpartum uterus (up to 48 h after delivery) and can be used alone as a desk-top simulator or placed inside the Mama Natalie for complete birth simulation. The Mama-U was designed to help fill a gap in many low- and middle-income countries: it provides a portable, low-cost simulation model for competency-based training that can be easily transported to the sites where female providers work. The Mama-U models tested in this study were produced by Laerdal outside of Pakistan and procured by Jhpiego.

Partner training institutions selected 10 qualified PPIUD trainers to conduct a standardized training that included the use of the Mama-U model. These trainers conducted seven three-day competency-based clinical trainings and a single one-day refresher update for midwives and doctors/WMOs. Participants were selected by partner organizations. Training details are presented in Table 1.

**Table 1:** PPIUD training conducted with the Mama-U model

Location	Type	Date	Participants
Greenstar Social Marketing, Lahore	1-day refresher	June 12, 2013	10
Pakistan Institute of Medical Sciences, Islamabad	3-day clinical training	June 19–21, 2013	15
Services Hospital, Lahore	3-day clinical training	June 25–27, 2013	10
District Headquarter Hospital, Mandi Bahauddin	3-day clinical training	July 15–17, 2013	10
National Committee for Maternal and Neonatal Health, Karachi	3-day clinical training	August 20–22, 2013	10
Lady Aitchison Hospital, Lahore	3-day clinical training	September 3–5, 2013	12
Lady Willingdon Hospital, Lahore	3-day clinical training	September 3–5, 2013	10
Services Hospital, Lahore	3-day clinical training	September 18–20, 2013	11
Total			88

A short-duration, blended-learning approach was used to improve providers’ clinical skills and confidence. The training covered counselling, medical eligibility criteria for family planning, PPIUD insertion procedures, management of side effects and complications, infection prevention, and routine follow-up for PPIUD clients. The intervention also included promotion of PPFP counseling at antenatal/postnatal clinics, provision of Mama-U models to each of the training center labor rooms, and integration of on-the-job coaching, follow-up, and monitoring into existing training programs.

### Data collection

#### Provider knowledge and clinical skills

Standard quality of care assessment tools were used to capture data on knowledge and clinical skills from all 88 providers who attended the PPIUD training sessions using the Mama-U model. The tools were developed by Jhpiego as part of a global learning resource package that is adapted as needed for individual country settings. The core checklist and questionnaire have been incorporated into national training packages; in India alone, close to 1.5 million PPIUD insertions have been carried out by providers trained using these materials.

The knowledge questionnaire included 25 questions, which were indexed to learning goals and objectives and topics covered during training (Additional file 2). The questionnaire was administered at the beginning and end of each training to assess changes in providers’ knowledge. The pre- and post-test ensured that learners and trainers understood what knowledge gaps existed and worked to eliminate them before the end of the course. The skills checklist was originally developed through an iterative desktop and clinical application process involving subject matter and instructional design experts and field-based trainers and providers (Additional file 1). The checklist is designed to be used both as a learning tool in the classroom and as an assessment tool in simulations and clinical practice. The final version of the checklist has been field tested and used in 26 countries where Jhpiego is implementing PPFP programming. The adaptation of the checklist used in this study was endorsed by the provincial Department of Health and the Population Welfare Department in Pakistan.

To assess the Mama-U trainings in Pakistan, assessors observed providers perform three tasks and completed the skills checklist; the tasks included counseling women on PPFP and the PPIUD during antenatal care, counseling women on PPFP and the PPIUD during early labor, and inserting a PPIUD in a client or Mama-U model. Providers were considered to have met clinical standards if they correctly performed 80% of the checklist items related to that task.

PPIUD insertions are difficult to schedule by the nature of the labor and delivery process. Therefore, the use of simulated as well as actual insertions in competency-based learning is an internationally recognized practice. Both methods were used in this assessment to measure provider performance. However, the eventual qualification and certification of providers to insert PPIUDs was based on demonstrable performance in clinical cases rather than simulation practice alone.

#### Training process

Following each training course, trainers completed a self-administered questionnaire that documented the number of participants and the extent to which the Mama-U model was used. All participants were asked to fill out an evaluation form, in which they rated different elements of the training process on a five-point scale and answered questions about their confidence level and prior training experiences. The forms were kept anonymous so that participants would openly share their views. Additionally, one FGD was conducted with all trainers following the completion of the trainings; they provided feedback on the appropriateness of the Mama-U model and suggestions for improvements.

#### Feasibility and acceptability of the Mama-U model

The benefits, risks, and acceptability of the Mama-U model for training and counseling clients were discussed during the FGD conducted with trainers and during two post-training FGDs conducted with providers in Islamabad (9 participants) and Mandi Bahauddin (10 participants).

#### Client perspectives

Structured individual interviews were conducted with 70 women who received PPIUDs, and two FGDs were held with 15 PPIUD clients. Designated focal persons at each study facility identified PPIUD clients, introduced the study, and collected contact information. A member of the study team then contacted each woman to arrange a time and place for the interview. Only clients who were 18–49 years old, had received a PPIUD within 48 h of delivery at a study facility, and consented to an interview were included in the study. FGD participants were selected from clients receiving PPIUDs as a part of two separate training sessions. FGD participants may have been contacted separately for an individual interview, but the files cannot be linked. During structured interviews, clients were asked about the counseling and clinical services they received from providers. FGDs explored community perceptions of PPFP and the PPIUD.

### Data analysis

Quantitative data were entered into SPSS Version 20 after each field activity and cross-checked to ensure their accuracy. Descriptive analysis was conducted to generate frequencies and proportions, and cross-tabulations were performed for various interlinked indicators. Differential analysis was conducted to create composite variables for pre- and post-training scores analysis.

Qualitative data from the FGDs and interviews were entered in MS Excel and coded to permit content analysis. A researcher summarized the prevalence of codes, identified similarities and differences in related codes across sources and contexts, and examined the relationship between codes. Themes and patterns were then identified and organized into emergent categories for discussion.

## Results

### Description of study participants

On average, the 10 trainers had 16.7 years of experience offering family planning services, including 2.7 years offering PPIUD services; 80% of trainers had no previous experience with the Mama-U model. Of the 88 providers who attended the training, 59 were doctors/WMOs and 29 served as midwives (13 LHVs, 12 nurses, 3 CMWs, and 1 FWW). LHWs were not included in this assessment as they are not currently allowed to provide clinical family planning services in Pakistan. The 19 providers who participated in FGDs included 11 doctors/WMOs and 8 midwives (3 nurses, 3 CMWs, and 2 LHVs). On average, FGD participants had 4.3 years of experience offering family planning services, including less than one year offering PPIUD services.

Individual interviews were conducted with 70 PPIUD clients. Interviewed clients were 28 years old, on average (range: 18–40 years), and 29% had primary education or less. Only 10% of respondents were interviewed after the birth of their first child; 70% of respondents had 2–4 children, including the one born prior to the interview. Less than one-fourth (22.9%) of respondents had used an IUD before. The average age of the 15 clients who participated in the FGDs was 26 (range: 20–31 years). Almost all (14) were housewives, 7 had 3–4 children, and 5 were having their first child.

### Effect of training on providers’ knowledge and skills

#### Knowledge test scores

Average scores on the 25-item knowledge test increased significantly after training for both doctors and midwives (Table 2). Although midwives had lower scores than doctors in the pre-test, their scores increased significantly more than doctors after training (by 14.3 versus 9.9 percentage points). Doctors and midwives had similar scores on the post-test, but the average score for both cadres was less than 80%.

**Table 2:** Change in knowledge scores after training, by cadre

Cadre	Average score (%)		Change from pre-test to post-test		*p*-value for difference between cadres
	Pre-test	Post-test	Percentage points	*p*-value	
Doctors/WMOs (*n* = 59)	69.8	79.7	+ 9.9	< 0.001	0.04
Midwives (*n* = 29)	62.8	77.1	+ 14.3	< 0.001	
*Total (n = 88*^*a*^*)*	*67.5*	*78.8*	*+ 11.3*		

^a^59 doctors and 29 midwives (including LHVs, CMWs, nurses and FWWs) were observed at the end of training; 19 doctors and 7 midwives (all were nurses) were observed at the two-month follow up

#### Skills assessment

At the end of training, observers found that about 70% of providers met the standards for PPFP/PPIUD counseling and 86.7% met the standard for inserting a PPIUD (Table 3). A higher proportion of doctors than midwives met each standard. Two months later, there were no significant changes, indicating that both doctors and midwives had retained these skills. Notably, the proportion of midwives who met the standard for PPFP/PPIUD counseling during antenatal care increased by 15 percentage points after training, a significantly greater change than the 1-percentage-point drop among doctors.

**Table 3:** Change in percent of providers who met standards from post-training to follow-up, by cadre

Standard and cadre	% of providers who met standard:		Change from post-training to follow-up		*p*-value for difference between cadres
	At end of training (*n* = 88)^a^	At two-month follow-up (*n* = 26)^a^	Percentage point change	*p*-value	
PPFP/PPIUD counseling during antenatal care					
Doctors	73.7	73.1	- 0.6	0.89	0.05
Midwives	60.9	75.9	+ 15.0	0.07	
Total	70.2	73.9	+ 3.7		
PPFP/PPIUD counseling during early labor					
Doctors	71.0	74.3	+ 3.3	0.61	0.59
Midwives	60.7	57.1	- 3.6	0.77	
Total	68.3	69.7	+ 1.4		
Insertion of PPIUD					
Doctors	87.9	83.8	- 4.1	0.21	0.64
Midwives	83.2	81.9	- 1.3	0.79	
Total	86.7	83.3	- 3.4		

^a^59 doctors and 29 midwives (including LHVs, CMWs, nurses and FWWs) were observed at the end of training; 19 doctors and 7 midwives (all were nurses) were observed at the two-month follow up

#### Client reports on counseling

According to client interviews, the majority of providers met most or all of the counseling requirements. However, midwives were almost five times more likely than doctors to meet all 10 key requirements during PPIUD counseling sessions (63.9% versus 13.3%, p = 0.004) (Table 4). When all 10 counseling requirements were met, clients were significantly more likely to be satisfied with the counseling (94% versus 52%, *p* < 0.05) and with their decision to have the PPIUD inserted (94% versus 71%, *p* < 0.05) (data not shown).

**Table 4:** Percent distribution of PPIUD counseling sessions by number of key requirements met, as reported by clients, according to cadre (*n* = 66^a^)

Number of counseling requirements met^b^	Sessions with doctors (*n* = 30)	Sessions with midwives (*n* = 36)	*p*-value
All (10)	13.3	63.9	0.004
Most (6–9)	46.7	33.3	
Half or fewer (1–5)	39.9	2.8	
*Total*	*100*	*100*	

^a^Four respondents were counseled on family planning by family members or others in the community rather than by a trained provider; they are excluded from the analysis

^b^Counseling requirements include: maintaining privacy, discussing future reproductive goals, discussing family planning options and choices, clearly understanding messages, allowed to ask questions, questions answered to satisfaction, information on PPIUD as long-acting method, information that PPIUD is non-hormonal, information that PPIUD does not require frequent clinic visits, and information on health timing and spacing

Twenty percent of clients interviewed said they were counseled using the Mama-U model; of these 14 clients, 79% said the demonstration helped them decide to accept the PPIUD (data not shown). Client FGDs provide an explanation for these results. During FGDs, clients reported that the demonstration helped them understand the placement of the PPIUD and removed concerns that it would move around in the abdomen and cause harm.

*“Yes, it [the model] was good. All the myths and fears of IUD are gone. After seeing it, now I am satisfied.”* (Mother of three sons, Lahore)

### Acceptability and feasibility of Mama-U model

#### Providers’ perceptions

As part of the training evaluation, the vast majority of providers agreed that the Mama-U model was easy to use and a good tool to teach insertion skills; about 70% strongly agreed with those statements (Table 5). Most providers also felt the model was helpful in addressing myths and misconceptions. Almost all (96.1%) of the providers recommended that the Mama-U model be used in future trainings (data not shown).

**Table 5:** Providers’ evaluation of the Mama U model (*n* = 88)

Item	Percent Distribution of Provider Responses			
	Strongly agree	Agree	Neutral	Disagree
The simulator was a good tool to teach this information	70.1	23.4	6.5	0
The simulator was easy to use	68.8	20.8	6.5	3.9
I had enough time to practice with the simulator	68.8	24.7	5.2	1.3
	Mostly	Somewhat	Not at all	Not applicable
Was this model helpful in addressing any myths and misconceptions you may have had about PPIUD?	70.1	20.8	3.9	5.2

**Fig. 2:**
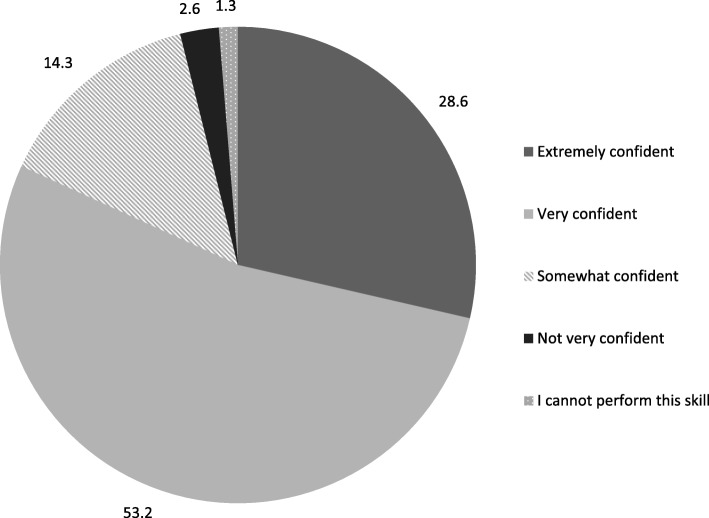
Providers’ confidence in providing PPIUD services after training (*n* = 88)

During FGDs, providers confirmed that the Mama-U model made it easy to learn the insertion procedure before working with real clients, and they highly recommended it for teaching and practice, including onsite refresher training. The model also built providers’ confidence in the technique. A postgraduate at PIMS noted that “confidence is achieved by practicing on this model,” and 81.8% of providers said they were extremely or very confident in their ability to provide PPIUD services after training with the Mama-U model (Fig. 2).

Providers also strongly supported the use of the Mama-U model for client counseling. Demonstrating the insertion process with the model helped the woman understand how and where the PPIUD would be placed and built her confidence in the method. Providers recommended designating a counselor to conduct this kind of counseling in the gynecology and obstetrics outpatient department.

#### Trainers’ perceptions

All trainers had experience with the anatomically realistic training model currently in use for instruction on PPIUD insertion. During FGDs, the trainers said they preferred the Mama-U model because it made the insertion technique clear, visible, and easy to explain; they also felt the uterovaginal angle’s clarity and closeness to reality and the position of the uterus were good. In contrast, they said the current model did not allow trainees to see the internal components while performing insertions and the cervix was hard to grasp.

Trainers noted several other logistical advantages of the Mama-U model, notably its low cost, light weight, and portability. The heavy, bulky, anatomically realistic model suffered by comparison:

*“Who will buy [the anatomically realistic model] worth 800 dollars? There is no comparison with [the anatomically realisticl] model. It is rigid and made of plastic.”* (Trainer, Lahore)

The trainers also observed that, in the Pakistan context, the abstract shape of the Mama-U was an advantage compared with the anatomical realism of the conventional model, because it reduces embarrassment among trainers, providers, and clients and allows the model to be used in patient waiting areas and other public spaces.

*“Before this model, I usually made different positions of my hands to make procedures understandable to the patient, but now I am using this model and it’s easy for me to make them understand.”* (Trainer, Lahore)

## Discussion

Expanding access to high quality PPFP services, including the PPIUD, in rural Pakistan requires closing the gaps in the availability of trained PPFP service providers. This will require midwives working at peripheral facilities and in communities to transition quickly from primarily acting as family planning counselors to independent service providers, without lengthy and costly trainings. This assessment aimed to determine if competency-based training using the Mama-U model can help meet this challenge. We assessed whether or not the training with the Mama-U model resulted in competent providers, who maintained their skills and knowledge for at least two months. Additionally, we assessed the acceptability of the Mama-U model to providers who participated in the training and who used the model post-training to maintain their skills. We found positive results for each of these assessments., indicating that this approach can be an effective, acceptable, and highly feasible way to teach providers the knowledge and skills needed to provide good quality PPIUD services. The model received positive reviews from trainers and providers, who made significant gains in knowledge during the trainings and generally mastered PPIUD insertion skills, although room for improvement exists. Another important benefit of using anatomic models for teaching and skills practice is their role in increasing provider confidence [17, 18], and this experience with the Mama-U bears that out. More than four-fifths of providers said they felt confident in their ability to provide PPIUD services after training.

Through follow-up assessments, this study also assessed retention of provider knowledge and skills. This expanded on existing research, which suggests that skill retention is improved by continued practice through access to and use of skills labs using simulators [19–21]. Notably, the low cost and portability of the Mama-U enables this kind of practice at facilities located far from training sites and a skills lab. This is important because experience shows that short duration, high frequency clinical skills training on simulators – backed by appropriate instruction and reinforcement – boosts confidence, improves clinical outcomes, and maintains skills [20, 21]. Regular onsite practice and refresher trainings may be especially important to maintain providers’ skills at rural facilities with low client loads, where providers have few opportunities to insert PPIUDs. A recent review of the literature concluded that simulation training has equal impact on individual learning regardless of whether it is conducted at an offsite training center or in the real-world environment of a health facility. However, the latter has the additional advantages of prompting organizational learning and enabling clinical teams to practice together [22].

Widely distributing the Mama-U model to skills labs and facilities has the potential to expand effective, competency-based training—and thus access to PPIUDs—to more remote areas while improving the quality of services. Increasing the number of simulation models in skills labs would allow more practice time for trainees, which has been shown to improve insertion outcomes [19] along with a range of obstetric and gynecological skills [20–22]. Keeping Mama-U models at facilities would permit onsite or on-the-job training, creating an opportunity to train providers where they work and adjust the training to address site- and area-specific challenges [21]. The models could also be used during supportive supervision and monitoring to help maintain providers’ skills.

The findings suggest that having the Mama-U on hand at facilities would also enable providers to use it as a job aid during PPIUD counseling. Trainers and providers noted that the model’s abstract design allows providers to demonstrate how the IUD is inserted in the body without embarrassing clients. Notably, family planning counseling during antenatal care was the only task in which providers’ skills improved during the follow-up period, suggesting that use of the Mama-U for training and counseling can help providers with family planning education and demand creation activities.

Finally, although the study did not explicitly seek to assess the appropriateness of task shifting, the findings support global recommendations that encourage task sharing and shifting for IUD services [23]. This kind of task shifting is especially important for Pakistan, given that those providing midwifery services (including nurses and community health workers) are far more accessible than doctors. Skills assessments found that those providing midwifery services were as likely as doctors to have good PPIUD insertion skills after training with the Mama-U and to retain them two months later. Clients reported that midwives (including community health workers) were five times more likely than doctors to meet all counseling requirements. This confirms existing research suggesting that a variety of health care cadres in resource-poor settings can be trained to safely and efficiently insert IUDs, including PPIUDs [13].

Although the results of this assessment are promising, they should be interpreted with care. Given the small sample size, it may not be appropriate to generalize the findings beyond the immediate context, especially given the loss to follow-up among clients, many of whom could not be traced or failed to return for follow-up. In the absence of a pre-training measure of providers’ clinical skills, it is also impossible to assess the effect of training on initial skills development. Instead, we focus on the ability of the approach to help providers retain skills over time and across provider types. Further research is required to examine provider skill retention beyond the two-month follow-up period in this study, but we feel that these short-term results are promising. It must also be acknowledged that deficiencies in provider skills remained after training. Finally, the study did not analyze the costs associated with various simulation models, but the market value of the Mama-U at the time of the study was approximately one-third the cost of the current simulation model. Further research is needed to confirm the cost benefits of using a Mama-U instead of the current simulation model, to identify areas of weakness in the training approach, and to explore links between the training approach, the Mama-U model, and improved clinical skills and services.

## Conclusion

Competency-based training with the Mama-U model in Pakistan has the potential to extend PPIUD service delivery to all those providing midwifery services in rural parts of the country (including nurses and various types of community health workers), while simultaneously improving the quality of counseling and insertion services. After training, these midwifery providers exceeded doctors/WMOs in counseling and equaled them in PPIUD insertion skills. However, the development of a durable, lightweight, low-cost simulation model has broader ramifications for how training is organized and delivered in low-resource settings. In Pakistan, the Mama-U will permit onsite, on-the-job training tailored to the local setting as well as the continuing practice that providers need to maintain their insertion skills, especially at facilities with low client volumes. The model’s acceptability as a counseling tool also presents an opportunity for providers to bolster family planning education and demand creation activities with clients and their families, contributing to increased use of PPFP and PPIUDs, a reduction in unmet need for family planning, and improvements in maternal and neonatal health outcomes.

## Additional files

Additional file 1: Performance Standards for PPIUD Counseling and Services. (PDF 775 kb)

Additional file 2: Postpartum IUD Knowledge Assessment Test. (PDF 287 kb)

## Data Availability

The datasets used and/or analyzed during the current study are available from the corresponding author on reasonable request.

## Abbreviations

CMW: Community Midwife
FGD: Focus Group Discussion
FWW: Female Welfare Worker
IUD: Intrauterine Device
LHV: Lady Health Volunteer
LHW: Lady Health Worker
PPFP: Postpartum Family Planning
PPIUD: Postpartum Intrauterine Device
WHO: World Health Organization
WMO: Woman Medical Officer
