# Supplementary material for: Reinvigorating postpartum intrauterine contraceptive device use in Pakistan: an observational assessment of competency-based training of health providers using low-cost simulation models
Source: BMC Med Educ. 2019 Jul 15;19:261. doi: 10.1186/s12909-019-1683-y (PMC6631998; doi:10.1186/s12909-019-1683-y)
Supplement: Supplementary file 1 — Performance Standards for PPIUD Counseling and Services. (PDF 775 kb) [file 12909_2019_1683_MOESM1_ESM.pdf]

Performance Standards for PPIUD Counseling and Services[a]

Performance Standards for PPIUD Counseling and Services			
**Number**	**Area**	**Performance Standards**	
		**Number**	**Total**
**1**	PPFP/PPIUD education and counseling and initial client screening during ANC; follow-up care/return visits	**1–8**	**8**
**2**	PPFP/PPIUD education and counseling and client assessment during early/inactive labor or the postpartum period	**9–3**	**5**
**3**	IUD insertion	**14–21**	**8**
**4**	Management of PPIUD services and recordkeeping	**22–26**	**5**

[a]Sources for these performance standards include the Jhpiego Family Planning Performance Standards for Afghanistan, the WHO/CCP's *Family Planning: A Global Handbook for Providers* and the Postpartum IUD training materials by Acquire/Engender Health.

FACILITY:____

ASSESSMENT TEAM:________________________ **DATE:**________________________

Performance Standards	Verification Criteria	Y/N, N/A[2]	Y/N, N/A	Comments
**Area 1: PPFP/PPIUD Education and Counseling and Initial Client Screening during ANC; Follow-Up Care/Return Visits**				
Instructions for the Assessor: Observe standards 1–6 in sequence with two women receiving PPFP counseling during an ANC visit. Observe provision of care to at least two women for standards 7 and 8.				
**1. Provider/counselor uses recommended counseling techniques for PPFP during ANC.**	**Observe in the appropriate clinical services area with client that the provider/counselor:**			
	• Shows respect for the woman and helps her feel at ease.			
	• Encourages the woman to explain needs, express concerns and ask questions.			
	• Includes the woman's husband or an important family member, with the woman's consent.			
	• Listens carefully.			
	• Respects and supports the woman's informed decisions.			
	• Checks to be sure the woman understands PPFP counseling messages.			
**2. Provider/counselor provides information on benefits of healthy pregnancy spacing (or limiting, if desired) and explores the woman's knowledge about (postpartum) family planning methods.**	**Observe that the provider/counselor:**			
	• Explores woman's knowledge about the benefits of pregnancy spacing.			
	• Asks about previous family planning methods used and knowledge about all family planning methods (LAM, progestin-only pills, postpartum ligation, condoms and the PPIUD).			
	• Addresses any related needs such as protection from STIs, including HIV and support for condom use.			

[2] Y = Yes; N = No; N/A = Not Applicable

Performance Standards	Verification Criteria	Y/N, N/A[2]	Y/N, N/A	Comments
Use the ***PPFP Counseling Job Aids*** (Appendix B) to facilitate this task.	• Corrects misinformation.			
	• Discusses the woman's situation, her plans and what is important to her about a method.			
	• Helps the woman consider suitable methods. If needed, helps her reach a decision.			
	• Supports the woman's choice.			
**3. Provider/counselor does a brief screening to determine whether the IUD is an appropriate method for the woman interested in a PPIUD.**	**If the woman is interested in the PPIUD, observe that the provider/counselor:**			
	• Determines that the woman does not have any of the following conditions:			
	• Malignant trophoblastic disease			
	• Cervical, endometrial or ovarian cancer			
	• Abnormalities of the reproductive tract/uterine fibroids that distort the uterine cavity			
	• Pelvic tuberculosis			
	• Increased personal risk of having gonorrhea or chlamydia infection			
	• AIDS and not clinically well or not on antiretroviral therapy			
	• If none of the above conditions are present, tells the woman that she is likely eligible to use the IUD.			
	• Proceeds with method-specific counseling for this method.<br><br>*[NOTE: The woman will be reassessed immediately postpartum for other conditions resulting from labor/delivery that may make the IUD a poor choice for her at this time.]*			

Performance Standards	Verification Criteria	Y/N, N/A[2]	Y/N, N/A	Comments
**4. Provider/counselor gives method-specific information about the IUD.**	**Observe that the provider/counselor:**			
	• Uses visual aids (poster, demonstration IUD) during counseling.			
	• Discusses key information with the woman:			
	• How effective the IUD is: prevents almost 100% of pregnancies			
	• How the IUD prevents pregnancy: causes a chemical change that damages the sperm BEFORE the sperm and egg meet			
	• How the IUD is used: inserted after delivery and then requires no additional care (Ensure that the woman knows it can be inserted at other times as well.)			
	• How long the IUD prevents pregnancy: up to 12 years (Copper T 380A)			
	• How the IUD can be removed at any time by a trained provider and fertility will return immediately			
	• Provides information about when the woman should come back.			

Performance Standards	Verification Criteria	Y/N, N/A[2]	Y/N, N/A	Comments
**5. Provider/counselor gives the woman more specific information about the PPIUD (e.g., advantages, limitations, when to return).**	**Observe that the provider/counselor:**			
	• Discusses the following advantages:			
	• Immediate placement after delivery			
	• No action required by the woman			
	• Immediate return of fertility upon removal			
	• Does not affect breastfeeding			
	• Long-acting and reversible: can be used to prevent pregnancy for a short time or as long as 12 years.			
	• Discusses the following limitations:			
	• Heavier and more painful menses, especially the first few cycles (may not be as noticeable to the postpartum woman because of the recovery process)			
	• Does not protect against STIs, including HIV			
	• Small risk of perforation			
	• Higher risk of expulsion when inserted postpartum (but this risk can be minimized through immediate [postplacental, intracesarean] insertion, using appropriate technique and instruments)			
	• Discusses the following warning signs and explains that the woman should return to the clinic as soon as possible if she has any of the following:			
	• Foul-smelling vaginal discharge, different from the usual lochia			
	• Lower abdominal pain, especially if accompanied by not feeling well, fever or chills			
	• Concerns that she might be pregnant			
	• Concerns that the IUD has fallen out			

Performance Standards	Verification Criteria	Y/N, N/A[2]	Y/N, N/A	Comments
**6. Provider/counselor annotates the woman's medical record to alert other care providers that she has chosen the PPIUD.**	**Observe that the provider/counselor:**			
	• Makes a notation of which PPFP method has been chosen.			
	• Documents on ANC record/card that the woman has been counseled and has requested the PPIUD.			
	• Instructs the woman that, when she comes in labor to deliver, she should tell the provider in the facility that she wants an IUD after delivery.			
	• Gives the woman the card that shows she has consented to postpartum insertion of the IUD.			
**Note:** In reality, between Steps 6 and 7, the women has had her baby, undergone AMTSL (following vaginal delivery), had an IUD inserted and been discharged from the facility with instructions to return at 6 weeks for routine follow-up, or whenever she has problems or concerns.				
**7. The provider conducts follow-up care/return visits appropriately.**	**Observe that the provider:**			
	• Greets the woman politely.			
	• Identifies the purpose of the visit.			
	• Ensures privacy and confidentiality.			
	• Allows the woman to ask questions.			
	• Asks if the woman has concerns or problems related to the IUD.			
	• Enquires about breastfeeding (if applicable).			
	• Asks the woman whether she has resumed sexual relations and whether she has concerns that she might be at increased risk of exposure to STI/HIV. Describes and offers condoms for dual protection, as appropriate.			
	• Where possible, performs pelvic examination and documents presence and length of string.			
	• Trims string, if appropriate or desired by the woman.			

Performance Standards	Verification Criteria	Y/N, N/A[2]	Y/N, N/A	Comments
	• Reminds the woman to return, if needed, and that she can have the IUD removed at any time at her request.			
	• Documents this and other information from visit in the chart.			
**8. The provider identifies women with problems and manages complications, as necessary.**<br><br>A more detailed discussion of management of side effects and complications is found in *Family Planning: A Global Handbook for Providers* (WHO and CCP 2007).	**Observe that the provider:**			
	• Asks the woman if she is experiencing any side effects or problems with the PPIUD.			
	• If side effects and/or problems are identified, conducts brief assessment and provides initial management: (noted here) and either manages accordingly or refers for additional treatment.			
	• *Heavy vaginal bleeding:* provides explanation and reassurance, assesses for anemia, performs pelvic exam, provides NSAIDs (ibuprofen 400 mg twice daily for 5 days), provides iron tablets. Aspirin should not be used because it has an anti-blood-clotting effect.			
	• *Irregular bleeding:* provides explanation and reassurance, provides NSAIDs (ibuprofen 400 mg twice daily for 5 days), provides iron tablets.			
	• *Low abdominal pain or cramping:* assesses for endometritis by palpating abdomen and observing vaginal discharge, provides explanation and reassurance, provides NSAIDs (ibuprofen 400 mg twice daily for 5 days).			
	• *Severe lower abdominal pain:* assesses for ectopic pregnancy or pelvic infection.			
	• *Fever and purulent vaginal discharge:* performs pelvic exam, assesses for pelvic infection. (Note: it is not necessary to remove the IUD during treatment)			
	• *Suspected pregnancy:* performs pelvic exam, assesses for pregnancy.			
	• *Suspected expulsion:* performs pelvic exam: if the IUD is partially expelled, removes and replaces it; if the IUD is not found, asks the woman if the IUD was expelled (offers replacement or another method); if the IUD is not found and the woman is unaware of expulsion, considers X-ray or ultrasound.			

Performance Standards	Verification Criteria	Y/N, N/A[2]	Y/N, N/A	Comments
	• *String problems:* too long—trims strings; not found—assesses for expulsion. Considers ultrasound to check location of the IUD.			
	• If initial management approaches are not effective, refers the woman for additional evaluation and management, as necessary.			
	• Offers to remove the IUD for any woman who requests to have it removed.			

Performance Standards	Verification Criteria	Y/N, N/A[2]	Y/N, N/A	Comments
**Area 2: IUD Counseling and Client Assessment during Labor or Postpartum Period**				
Instructions for the Assessor: Observe provision of service to at least one woman for each of standards 9, 10 and 11. Observe provision of care to at least two women for standards 12 and 13.				
**9. The provider re-confirms with the laboring woman that she has chosen the IUD for postpartum family planning.**	**Observe that the provider:**			
	• Greets the patient (and her companion, if present) with respect.			
	• Introduces self to the patient (and her companion, if present).			
	• Confirms the patient identifier information (name, date of birth).			
	• If the woman is in labor, is sensitive to the woman's discomfort and pauses the discussion during contractions/labor pains.			
	• Determines, using the ***Pre-Insertion Screening Job Aid***, that the woman meets criteria for postplacental insertion.			
	◦ Has had family planning counseling when not in active labor.			
	◦ Has indicated consent.			
	◦ Insertion can occur immediately following delivery.			
	• Determines that the IUD is appropriate for the woman (see Standard 12) and that she still desires the IUD.			

Performance Standards	Verification Criteria	Y/N, N/A[2]	Y/N, N/A	Comments
**10. The provider re-confirms with the postpartum woman that she has chosen the IUD for PPFP.**	**Observe that the provider:**			
	• Greets the patient (and her companion, if present) with respect.			
	• Introduces self to the patient (and her companion, if present).			
	• Confirms the patient identifier information (name, date of birth).			
	• Determines that the woman meets criteria for postplacental insertion.			
	◦ Has had family planning counseling when not in active labor.			
	◦ Has indicated consent.			
	• Determines, using the ***Pre-Insertion Screening Job Aid***, that the IUD is appropriate for the woman (see Standard 12) and that she still desires the IUD.			

Performance Standards	Verification Criteria	Y/N, N/A[2]	Y/N, N/A	Comments
**11. The provider counsels and screens a woman who was not identified during ANC for the PPIUD.**	**Observe that the provider:**			
	● Identifies laboring and postpartum women who are interested in the PPIUD.			
	● If the woman is in early labor or postpartum, ensures woman is comfortable and capable of making an informed choice.			
	● Performs a brief screening assessment and determines whether the PPIUD is an appropriate method for the woman (see Standard 3).			
	● Provides method-specific information about the PPIUD (see Standards 4 and 5).			
	● Makes a notation in the medical record and notifies other care providers that the woman has chosen postpartum insertion of the IUD.			
	● Where appropriate for the postpartum woman or the woman who has been unable to have postplacental insertion, makes arrangements for early PPIUD insertion before discharge.			
**12. The provider ensures the IUD is an appropriate postpartum contraceptive method for a laboring/recently postpartum woman.**	**Observe that the provider:**			
	● Uses the ***Pre-Insertion Screening Job Aid*** to ensure that none of the following medical conditions are present:			
	● Postpartum endometritis/metritis			
	● Puerperal sepsis			
	● More than 18 hours from rupture of membranes to delivery of the baby			
	● Unresolved postpartum hemorrhage			
	● Extensive genital trauma where the repair would be disrupted by postpartum placement of the IUD			

Performance Standards	Verification Criteria	Y/N, N/A[2]	Y/N, N/A	Comments
**13. The provider demonstrates good client-provider interaction.**	**Observe that the provider:**			
	● Uses the patient's name, as appropriate for the setting.			
	● Provides the patient with an opportunity to ask questions; answers the patient's (and if present, her companion's) questions.			
	● Maintains privacy and confidentiality for the woman.			
	● Demonstrates active listening.			
	● Speaks respectfully and professionally with the patient in clear and simple language.			
	● Ensures that the patient understands the information provided.			
**Area 3: IUD Service Provision**				
Instructions for the Assessor: Observe the provision of IUD services to at least two women each for standards 14–21. If there are no women, have providers demonstrate service provision on anatomic models, AND review the clinical record of the two most recent cases of each type of service provision (postplacental, intracesarean and early postpartum). Cases should not be more than 6 months old.				
**Immediate PPIUD Insertion**				
**14. The provider completes all <u>pre-insertion tasks</u> for postplacental or intracesarean IUD insertion.**<br><br>Use the ***Pre-Insertion Screening Job Aid*** to help facilitate this task.	**Observe that the provider:**			
	● Ensures that the woman has consented to PPIUD insertion.			
	● Ensures that the needed supplies and equipment are available in the room.			
	For postplacental insertion:			
	● Long placental forceps for insertion			
	● Ring forceps for grasping the cervix			
	● Retractor or Simms speculum			
	● Gauze pads/cotton balls			
	● Betadine			
	For intracesarean insertion:			

Performance Standards	Verification Criteria	Y/N, N/A[2]	Y/N, N/A	Comments
	• Ring forceps for inserting the IUD			
	• Opens the IUD onto a sterile delivery tray (postplacental) or an instrument tray (intracesarean).			
