# Supplementary material for: Reinvigorating postpartum intrauterine contraceptive device use in Pakistan: an observational assessment of competency-based training of health providers using low-cost simulation models
Source: BMC Med Educ. 2019 Jul 15;19:261. doi: 10.1186/s12909-019-1683-y (PMC6631998; doi:10.1186/s12909-019-1683-y)
Supplement: Supplementary file 2 — Postpartum IUD Knowledge Assessment Test. (PDF 287 kb) [file 12909_2019_1683_MOESM2_ESM.pdf]

Postpartum IUD Knowledge Assessment Test

Decide whether each of the following statements is T (true) or F (false). Write your answer in the space provided for each statement.

Postpartum IUD Overview

1._____ Postpartum contraception helps couples practice healthy spacing of pregnancies.

2._____ The most appropriate timing for postpartum IUD insertion is between 48 hours and four weeks postpartum.

3._____ Complaints about bleeding after postpartum IUD insertion are reported to be less than those for interval insertion.

Postpartum Anatomy and Physiology

4._____ Immediately after the placenta is expelled; the cervix and lower uterine segment are collapsed and limp.

5. _____ The immediate postpartum uterus is a smooth cavity with narrow apposition of the anterior and posterior walls, each of which is 4-5 cm thick.

6._____ In the immediate postpartum woman, the lower uterine segment is contracted, and slight pressure with the forceps is needed to move the IUD to the fundus.

Counseling and Informed Choice

7._____ An IUD should not be inserted postpartum if the client has not been counseled.

8._____ The best time to counsel a client for postpartum family planning is immediately following delivery.

9._____ It is important to inform clients that during the first follow-up visit, the possibility of "missing strings" is higher for postpartum IUD insertion than for interval IUD insertion.

Client Assessment

10. _____ A general medical and obstetric history, a sexually transmitted infection (STI) risk assessment; and a confirmation of marital status are essential components of a client history for a postpartum IUD candidate.

11. _____ Prolonged rupture of membranes or prolonged labor could increase the risk of infection; the provision of an IUD postpartum might need to be postponed.

12. _____ If a complete client history has been taken, the provider does not need to perform a post delivery physical exam before the IUD is inserted postpartum.

Infection Prevention

13. _____ The best way to prevent infections at a health facility is by following standard precautions.

14. _____ Decontamination and cleaning of the table top are necessary at the end of each day, not in between clients.

15. _____ When using the "no-touch" technique, if the IUD is inserted into the uterus and then removed back through the cervix, it cannot be reinserted through the cervix once again.

Postpartum IUD Insertion Techniques

16. _____ Postplacental insertion should take place within 10 minutes after expulsion of the placenta following a vaginal delivery.

17. _____ There is the same probability of IUD expulsion after a ringed forceps postplacental insertion as after a ringed forceps immediate postpartum insertion.

18. _____ A forceps insertion could be easier to perform in client whose uterus has contracted due to the active management of the third stage of labor.

19. _____ Anesthesia in addition to that which is given during delivery is required for postpartum IUD insertion.

Postpartum IUD Follow-Up

20. _____ Pain with intercourse is a common side effect of postpartum IUD insertion.

21. _____ Regardless of the reason, if the client requests it, the IUD should be removed.

22. _____ If the IUD strings are not visible at the first routine follow-up visit after a postpartum insertion, expulsion has definitely occurred.

Prevention and Management of Side Effects and Complications

23. _____ Bleeding and cramping side effects may not be attributed by the client to the postpartum IUD, since these are characteristic of the uterus's postpartum involution.

24. _____ The risk if expulsion after postpartum IUD insertion is minimal.

25. _____ Some times during the first postpartum IUD post insertion visit, the strings may have not yet descended.
